# THE DEVELOPMENT OF AN INTERVENTION TO IMPROVE SHARED DECISION MAKING IN GERIATRIC OUTPATIENTS

**DOI:** 10.1093/geroni/igz038.1091

**Published:** 2019-11-08

**Authors:** Ruth E Pel-Littel, Julia van Weert, Mirella Minkman, Wilma Scholte op Reimer, Marjolein van de Pol, Bianca Buurman

**Affiliations:** 1 Vilans, Utrecht, Utrecht, Netherlands; 2 Amsterdam School of Communication Research/ASCoR, University of Amsterdam, Amsterdam, Netherlands; 3 Vilans, Utrecht, Netherlands; 4 ACHIEVE, Centre of Applied Research, Faculty of Health, Amsterdam University of Applied Sciences, Amsterdam, Netherlands; 5 Department of Primary and Community Care, Radboud University Medical Center, Nijmegen, Netherlands; 6 Department of Internal Medicine, Section of Geriatric Medicine, Academic Medical Centre, University of Amsterdam, Amsterdam, Netherlands

## Abstract

Shared decision making (SDM) contributes to personalised decisions that fit the personal preferences of patients. However, older adults frequently face multiple chronic conditions (MCC). Therefore, implementing SDM requires special features. The aim of this paper is to describe the development of an intervention to improve SDM in older adults with MCC. Following the Medical Research Council framework for developing complex interventions, the SDMMCC intervention was developed step-wise. Based on a literature review and empirical research we developed in a co-creation process with the end-users a training for geriatricians and a preparatory tool for older patients with MCC and informal caregivers. After assessing feasibility the intervention was implemented at two outpatient geriatric clinics in a pilot study (N=108). Key elements of the training for geriatricians include: developing skills how to involve older adults with MCC and informal caregivers in SDM and learning how to explore personal goals related to quality of life. Key elements of the preparatory tool for patients include: an explicit invitation to participate in SDM, nomination that the patient’s own knowledge is valuable, invitation to form a partnership with the geriatrician, encouragement to share information about daily and social functioning and exploration of possible goals. Furthermore, invitation of informal caregivers to share their concerns. Through a process of co-creation both a training for geriatricians and a preparatory tool for older adults and their informal caregivers were developed, tailored to the needs of the end-users and based on the ‘Dynamic model of SDM with frail older adults’.

